# Armt1: a phoenix rises from the ashes

**DOI:** 10.18632/oncotarget.5964

**Published:** 2015-10-04

**Authors:** Derek Hoelz

**Affiliations:** Department of Basic Pharmaceutical Sciences, Husson University School of Pharmacy, Bangor, ME, USA

**Keywords:** Pathology Section, protein methylation, DNA damage response

In the mid 1960s scientists discovered an enzyme activity in pituitary extracts that hydrolyzed *s*-adenosyl methionine (AdoMet) leading to the generation of methanol. Initially, the sole purpose for this enzyme was thought to be cleavage of AdoMet into methanol; but, it was later identified as protein carboxyl methyltransferase or protein methylase II [1]. Similar to the already described protein methylase I, protein methylase II required the co-factor AdoMet to donate its methyl group. But unlike protein methylase I, protein methylase II did not N-methylate amine groups of lysyl and arginyl side chains. Instead, it appeared to O-methylate free carboxyl groups in aspartyl and glutamyl residues and/or the carboxyl-termini of proteins. The exact target(s) of protein methylase II and the significance O-methylation had on protein function was unclear.

Some years later an important role for this type of enzyme was described in chemotactic bacteria. In 1977, Springer and Koshland, Jr. uncovered that a necessary component of the bacterial chemical sensing system and product of the *CheR* gene was a protein O-methyltransferase [2]. It is now known that methylation of four specific glutamyl residues in chemoreceptors by CheR is required for adaption in chemotaxis for some bacterial species. It was logical that a system similar to CheR-regulated bacterial chemotaxis would be present in higher organisms. Despite studies identifying protein O-methyltransferase activities in cytoplasmic membranes of mouse neutrophils and mammalian targets of protein methylase II being erythrocyte membrane proteins and the pituitary hormone ACTH, a direct link between protein methylase II and eukaryotic cell signal transduction would not be made.

In the early 1980s, O'Connor and Clarke challenged the idea that protein methylase II was involved in signaling events in eukaryotic cells when they discovered that the mammalian enzyme catalyzed the transfer of methyl groups from AdoMet not to glutamyl or aspartyl residues, but to damaged L-isoapartyl or D-aspartyl residues that occur spontaneously in aging proteins [3]. Aswad subsequently demonstrated that methylation of ACTH by protein methylase II required deamination of an asparginyl residue in the hormone [4]. Protein methylase II hence would come to be known as the protein repair enzyme protein L-isoaspartate (D-aspartate) O-methyltransferase 1 (PCMT1). Two additional protein carboxyl methyltransferases would be identified in the 1990s: isoprenylcysteine carboxyl methyltransferase (ICMT) and leucine carboxyl methyltransferase (LCMT1), but neither of these enzymes would target glutamyl or undamaged aspartyl residues. It seemed that a eukaryotic equivalent of the prokaryotic glutamyl methyltransferase CheR was either very elusive or did not exist.

A significant challenge to describing a eukaryotic glutamyl methyltransferase was a lack of identified protein targets of this activity. Compared to N-methylation, protein O-methylation is inherently unstable and readily hydrolyzes at physiological pH making identification difficult. However, advances in protein mass spectrometry techniques and instrumentation now permit the detection of residual protein O-methylation. In 2006, we reported the LC-MS/MS sequencing and post-translational state of the DNA sliding clamp proliferating cell nuclear antigen (PCNA) in breast cancer cells [5]. PCNA has critical roles in the DNA damage response and its post-translational modification is a crucial regulator of its DNA replication and repair functions [6]. For example, ubiquitination and sumoylation of PCNA directs it to post-replication DNA repair, and tyrosine phosphorylation of PCNA by EGFR stabilizes the chromatin-bound form of the protein preventing its proteosomal degradation. We therefore carefully scrutinized PCNA from breast cancer cells for additional post-translational modifications and observed an interesting result - O-methylation or methyl esterification of glutamyl and aspartyl residues. Although plausible that aspartyl residue methylation was the product of PIMT-dependent protein repair activity, the overwhelming majority of methylation occurred on glutamyl residues [5]. To our knowledge, this was the first glutamyl methylation of a eukaryotic protein identified via mass spectrometry. And this discovery indicated that a glutamyl methyltransferase existed in eukaryotes after all.

Following this discovery, we examined breast cancer cell extracts for AdoMet-dependent O-methyltransferase activity and established PCNA as a specific target of this enzymatic activity [7]. We next identified the uncharacterized product of the *C6orf211* gene as a potential O-methyltransferase. Intriguingly, the C-terminus of C6orf211 possessed a highly conserved ‘domain of unknown function’ (DUF89). Examination of this domain identified it as a potentially unique methyltransferase fold with structural similarities to the AdoMet binding pocket of the prokaryotic glutamyl methyltransferase CheR. *In vitro* this gene product was capable of O-methylating PCNA, and unexpectedly, activity was observed in the absence of PCNA supporting its ability to O-methylate itself. Furthermore, our data supported automethylation as inhibitory, which helps explain why this activity had been so difficult to isolate in the past. After nearly 50 years, we assert that a CheR-like methyltransferase does exist in eukaryotic cells. The prototypical enzyme, acidic residue methyltransferase 1 (Armt1), has a vital role in regulation of the DNA damage response in human cells, which is likely conveyed through its ability to O-methylate glutamyl residues of the DNA repair factor PCNA.
